# Development of a Simple Mechanical Screening Method for Predicting the Feedability of a Pharmaceutical FDM 3D Printing Filament

**DOI:** 10.1007/s11095-018-2432-3

**Published:** 2018-05-31

**Authors:** Jehad M. Nasereddin, Nikolaus Wellner, Muqdad Alhijjaj, Peter Belton, Sheng Qi

**Affiliations:** 1 School of Pharmacy, University of East Anglia, Norwich, Norfolk, NR4 7TJ UK; 2 Norwich Research Park, Quadram Institute Bioscience, Colney Norwich, Norfolk, NR4 7UA UK; 3 Department of Pharmaceutics, College of Pharmacy, University of Basrah, Basrah, Iraq; 4 School of Chemistry, University of East Anglia, Norwich, Norfolk, NR4 7TJ UK

**Keywords:** feedability screening, fused deposition modeling 3D printing, hot melt extrusion, plasticization, printability, solid dispersions

## Abstract

**Purpose:**

The filament-based feeding mechanism employed by the majority of fused deposition modelling (FDM) 3D printers dictates that the materials must have very specific mechanical characteristics. Without a suitable mechanical profile, the filament can cause blockages in the printer. The purpose of this study was to develop a method to screen the mechanical properties of pharmaceutically-relevant, hot-melt extruded filaments to predetermine their suitability for FDM.

**Methods:**

A texture analyzer was used to simulate the forces a filament is subjected to inside the printer. The texture analyzer produced a force-distance curve referred to as the flexibility profile. Principal Component Analysis and Correlation Analysis statistical methods were then used to compare the flexibility profiles of commercial filaments to in-house made filaments.

**Results:**

Principal component analysis showed clearly separated clustering of filaments that suffer from mechanical defects *versus* filaments which are suitable for printing. Correlation scores likewise showed significantly greater values with feedable filaments than their mechanically deficient counterparts.

**Conclusion:**

The screening method developed in this study showed, with statistical significance and reproducibility, the ability to predetermine the feedability of extruded filaments into an FDM printer.

**Electronic supplementary material:**

The online version of this article (10.1007/s11095-018-2432-3) contains supplementary material, which is available to authorized users.

## Introduction

**Fig. 1:**
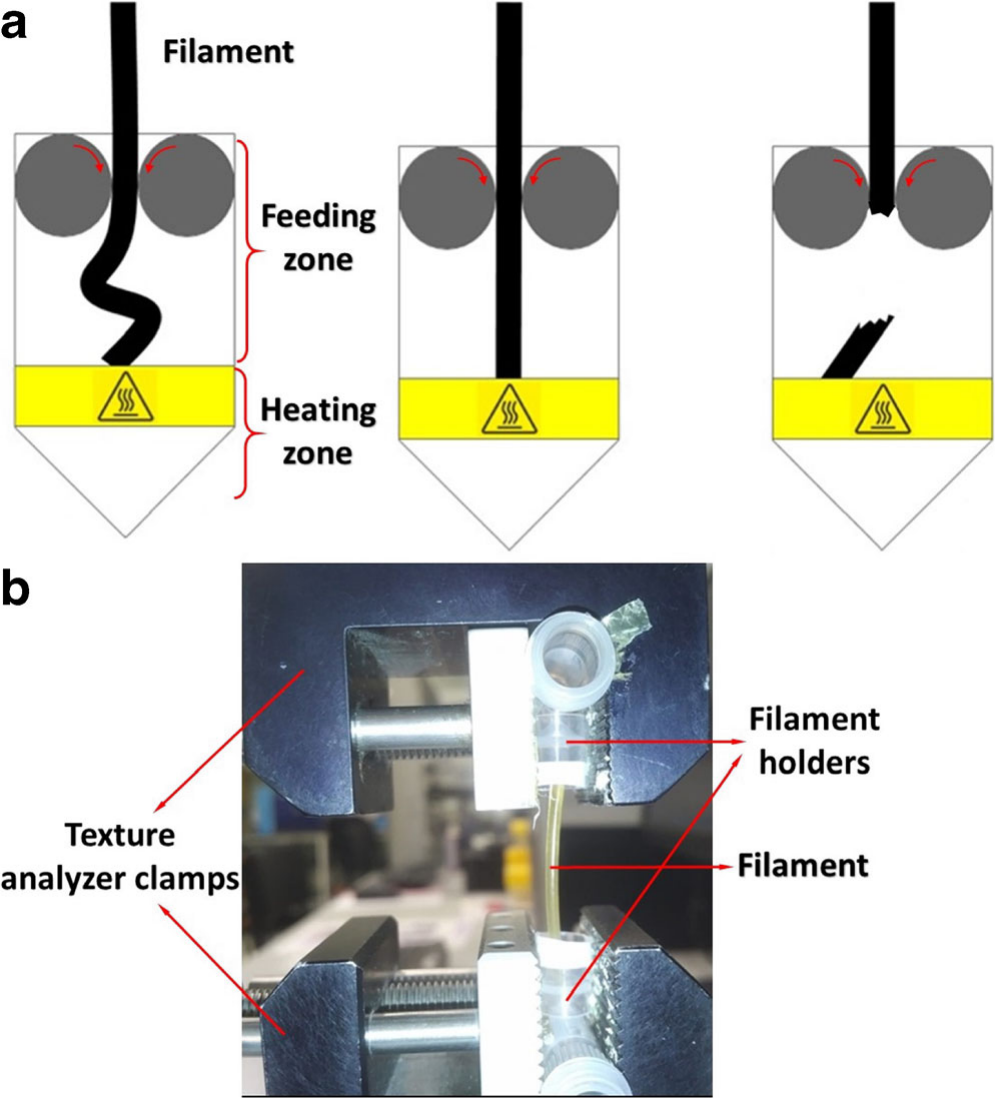
(**a**) Illustration of the different behaviour of filaments during feeding; (**b**) the texture analysis filament feedability test rig.

In recent years, there has been a rise in interest in utilizing 3D printing (3DP) as means to manufacture pharmaceutical dosage forms due to its ability to produce bespoke objects possessing high geometrical complexity quickly with high precision and accuracy. This gives 3DP the potential for the manufacturing of personalized dosage forms (1,2). FDM is a variant of 3DP that utilizes filament-shaped thermoplastic polymers as the building material. The mechanical assembly of a FDM printing head consists of the feeding rollers, heating zone, and the nozzle (3–5). The filament is fed into the printer by the action of the two counter-rotating feeding rollers, as illustrated in Fig. 1a. The filament is fed to a heating zone where the filament is melted and extruded through the printing nozzle onto the build plate layer-wise to form the desired object.

For pharmaceutical applications of FDM, the incorporation of a drug substance into the filament is achieved by two methods, impregnation and hot melt extrusion (HME) (2). Impregnation is done by immersing the filament in an organic solution of the drug. This method often yields low levels of drug loading (6–8). Preparation of FDM filaments by HME is the more attractive method for pharmaceutical applications as it allows for higher drug loading than the impregnation method, and allows for the use of pharmaceutical-grade polymers (9–11). Most hot melt extrudable pharmaceutical grade polymers, however, do not possess the required properties to allow for good quality FDM printing. This has been recognized as a significant technical barrier for further developing the pharmaceutical applications of FDM printing (10).

Critical process parameters (CPPs) which govern FDM are categorised into three types of parameters: machine-specific, operation-specific, and material-specific parameters (12). Machine-specific parameters are parameters relating to the make and model of the printer, operation-specific parameters are parameters relating to the processing conditions during the printing run, and material-specific parameters are parameters relating to the physiochemical properties of the material being printed. Most studies reported in the literature utilize commercially available, hobbyist application printers which do not allow for control of the machine-specific parameters. While such printers allow control over operation-specific parameters such as printing speed and temperature, restrictions imposed by polymer melt rheology and the printer feeding mechanism generally result most pharmaceutical polymers being unsuitable for FDM applications. Although the CPPs of FDM have been identified and are relatively well understood, there exists, to the best of our knowledge, no preformulation tools have been developed to allow for a quick and rational design of pharmaceutical FDM formulations. Most formulations reported in the literature employed a trial-and-error approach for development.

To achieve a continuous, high-throughput FDM printing operation, the filament needs to be firstly feedable through the feeding zone of the printer, and must also possess suitable melt flow properties to be printable once is transferred into the heating zone of the printing head. The filament-based feeding mechanism employed by FDM printers utilizes mechanical gearing arrangements to push the filament into the heating zone (Fig. 1a) (4). In order to accurately control the feeding rate, the filament has to be held tightly (pinched) between the two rollers (13), leaving it effectively under compression throughout the feeding process. In situations where the filament is brittle, this is likely to cause the filament to fracture, discontinuing the forward propulsion of the filament, causing a blockage in the printing head, as illustrated in Fig. 1a. Blockages in the printing head are very problematic; broken pieces of filaments inside the printing head can contaminate the machine, compromising the purity of any dosage forms one wishes to fabricate. Blockages in the printing head are also difficult to clean, often requiring disassembly of the entire printing head to be cleared. Therefore, there is a need for screening the mechanical suitability of the filament before attempting to feed the filament into a printer.

For the scope of this article, the term *feedability* is used to describe the mechanical suitability of a filament for FDM, with non-feedable filaments being filaments that can cause a block in the printing head of an FDM printer, regardless of whether their melt flow rheology is within acceptable limits for FDM. This study describes the development of a new formulation screening tool for the predetermination of the feedability of FDM filaments fabricated by HME from pharmaceutically relevant polymers. This tool was built based on the understanding of the relationship between the mechanical properties of some pharmaceutical polymer blends and how they correlate with their suitability for FDM. A number of simple in-house filaments, commercial filaments, as well as a printable complex pharmaceutical filament previously reported in the literature (the material was a placebo filament containing Eudragit EPO, Tween 80, PEG and PEO named as EUD was used in this study) (10) were prepared and attempted to be fed into an unmodified, commercial 3D printer to determine their feedability. A custom-made texture analyzer rig was used to test the filament response in a compress-and-release cycle, yielding a plot of force (exerted by the filament as resistance to deformation) *vs*. distance compressed plot. The texture analysis experiments were used to quantify the mechanical properties of the filaments and to ascertain whether it is possible to predetermine the feedability of a filament without having to compromise the printing head.

Using the force/distance plots (hereinafter referred to as the *flexibility profile*) produced by commercial filaments as a control, correlation analysis and principle component analysis (PCA) were used to determine whether there exists a statistically significant correlation between the flexibility profiles of different filaments and their feedability (and subsequently printability). This allows one to predetermine if hot melt extruded filament possesses adequate mechanical properties to be feedable. A test which can be used as a performulation tool to minimize trial-and-error when developing pharmaceutical formulations for FDM. The relationship between the formulation composition and the feedability of the filaments investigated in this study can bring new insights into the development of principles in rationalization of FDM formulation design.

## Materials and Methods

### Materials

Acrylonitrile butadiene styrene (ABS) and Makerbot^®^ Dissolvable Filament commercial filaments were purchased from Makerbot (Makerbot Industries LLC., New York, United States). Polylactic acid (PLA) commercial 3D printing filaments were purchased from XYZprinting (XYZprinting Inc., California, United States). All three commercial filaments were used as purchased. Pre-plasticized polyvinyl alcohol (otherwise known as Mowiflex^®^) C-17 grade pellets were graciously donated by Kurary (Kurary GmbH, Frankfurt, Germany). Hypromellose acetate succinate (HPMCAS, low-fine grade) was graciously donated by Shin Etsu (Shin Etsu Inc., Tokyo, Japan). Polysorbate (Tween^®^ 80) was purchased from Acros Organics (Acros Organics, Geel, Belgium). Polyethylene oxide N-10 grade (PEO; molecular weight = 100,000) was graciously donated by Colorcon (Colorcon Ltd., Dartford, United Kingdom). Eudragit® EPO was graciously donated by Evonik industries (Evonik, Darmstadt,Germany). Soluplus^®^ and Kollidon^®^ vinyl acetate 64 (polyvinyl pyrrolidone vinyl acetate 64) were graciously donated by BASF (BASF inc., Ludwigshafen, Germany). Polyethylene glycol 4000 (PEG; molecular weight = 4000) and paracetamol (PAC) were purchased from Sigma Aldrich (Sigma Aldrich, Salisbury, United Kingdom).

### Preparation of In-House Filaments

In-house filaments were prepared by HME, using a Haake Minilab II hot melt compounder (Thermo Fisher Scientific, Karlsruhe, Germany) equipped with a 1.75mm circular die. A list of prepared formulations and their key extrusion parameters can be found in Table I. All multi-component formulations were cycled in the extruder for 5 min at a screw speed of 100 RPM to ensure homogenous mixing (10). Following extrusion, filaments with diameters of 1.75mm ± 0.05 mm were collected for further testing.

**Table I:** Compositions and Extrusion Conditions of the Filaments

Formulation	Constituents	Extrusion temperature
Mowiflex®	Mowiflex® (contains PVA and an undeclared plasticizer at an undeclared concentration	170°C
HPMCAS	HPMCAS (100% *w*/w)	170°C
PEO	PEO (100% w/w)	75 °C
PVP/VA 64	PVP/VA 64 (100% w/w)	140°C
Soluplus®	Soluplus® (100% w/w)	120°C
Eudragit® EPO	Eudragit® EPO (100% w/w)	120°C
EUD	Eudragit EPO (55.5% w/w) + 11.1% *w*/w, 16.7% w/w, and 16.7% Tween 80, PEG, and PEO, respectively	100 °C
HD	HPMCAS (90% w/w) and paracetamol (10% *w*/w)	150°C
HP10	HPMCAS (90% w/w) and PEO (10% w/w)	150°C
HP10D	HPMCAS (81% w/w), PEO (9% w/w) and paracetamol (10% w/w)	140°C
HP20	HPMCAS (80% w/w) and PEO (20% w/w)	150°C
HP20D	HPMCAS (72% w/w), PEO (18% w/w) and paracetamol (10% w/w)	130°C
HP30	HPMCAS (70% w/w) and PEO (30% w/w)	140°C
HP30D	HPMCAS (63% w/w), PEO (27% w/w) and paracetamol (10% w/w)	120°C
HP40	HPMCAS (60% w/w) and PEO (40% w/w)	130°C
HP70	HPMCAS (30% w/w) and PEO (70% w/w)	100 °C
HP90	HPMCAS (10% w/w) and PEO (90% w/w)	85°C
SP	Soluplus® (90% w/w) and PEG (10% w/w)	110°C
ST	Soluplus® (80% w/w) and Tween® 80 (20% w/w)	100 °C

### Filament Characterization

#### Differential Scanning Calorimetry (DSC)

DSC was conducted using a Q20 differential scanning calorimeter (TA Instruments, Newcastle, United States). All in-house prepared filaments were tested using a heat-cool-reheat cycle with a temperature range of 20°C to 185°C at 10°C/min. All samples were tested as fresh samples immediately after extrusion. All tests were done in triplicates.

### Attenuated Total Reflectance Fourier Transform Infrared Spectroscopy (ATR-FTIR)

FTIR was conducted using a Vertex 70 FTIR spectrometer (Bruker Optics Ltd., United Kingdom), equipped with a MIRacle™ single reflection ATR accessory (Pike Technologies, United States) fitted with a diamond internal reflection element. ATR-FTIR spectra were acquired in absorbance mode, using a resolution of 2 cm^−1^, 32 scans for each sample, within the range of wavenumbers from 4000 to 550 cm^−1^. Spectra analysis was conducted using OPUS version 7.8 (Bruker Optics Ltd., United Kingdom). All measurements were done in triplicate.

### Powder X-Ray Diffraction (PXRD)

A Thermo ARL Xtra X-ray diffractometer (Thermo Scientific, Switzerland) equipped with a copper X-ray Tube (k = 1.540562 Å) was used to detect the presence of drug crystals (if any) in the extruded filaments. A scanning range of 3° < 2θ < 30°, using a step scan mode with step width of 0.01° and scan speed of 1 s/step was used to conduct all measurements.

### FDM Feedability Testing

Feedability of the extruded filaments was tested by feeding into a standard Makerbot® Replicator 2X commercial FDM 3D printer (Makerbot Industries LLC., New York, United States). Successful extrusion of the polymer through the nozzle tip was regarded as successful feeding, making the filament *feedable*. It should be highlighted that the printing quality was not assessed and is out of the scope of this study. All filaments were fed at the printer’s default printing temperature of 230°C.

### Texture Analysis (TA) Screening Test

Compression tests that simulate the feeding process of the filament through the printing head were performed using a TA.XT2 Plus Texture Analyzer (Stable Micro Systems, Godalming, United Kingdom) equipped with an in-house rig (Fig. 1b) and a 5 kg Load cell. Filaments were compressed axially with a compression speed of 3.15 mm/sec, corresponding to the roller movement speed of a Makerbot® Replicator 2X (determined by feeding an accurately cut 10 cm filament into the printer head and measuring the time needed for the filament to pass through the printing head). 5 cm long filament pieces were held standing in conical end caps to allow bending and to avoid fracturing them with the clamps. The compression distance was set to 15 mm with a trigger force of 0.05 N and data was collected during both compression and release. TA tests were done in triplicate for all tested filaments.

### TA Data Manipulation and Statistical Analysis

Since the materials tested were of varying hardness, the scaling of the flexibility profile (force (N) *vs*. distance (mm)) plots was not directly comparable without data range normalization. Therefore, data range normalization was performed using the equation

1 $$ {Y}_{Normalized}^n=\frac{Y^n}{\Sigma Y} $$

where Y^n^ is the n^th^ point on the Y-axis (force). Correlation analysis of the flexibility profiles of the pharmaceutical filaments with those of the commercial filaments was conducted using Microsoft® Excel 2016 expanded with the Data Analysis add-on. PCA was conducted using IBM® SPSS Statistical Analysis Suite (version 25), with the Varimax rotation method, 25 iterations for convergence, extracting components possessing an eigenvalue ≥1. The loadings of the three components with an eigenvalue at or above 1 are reported in Table S1 in the Supplementary Materials.

## Results

### Filament Characterization

**Fig. 2:**
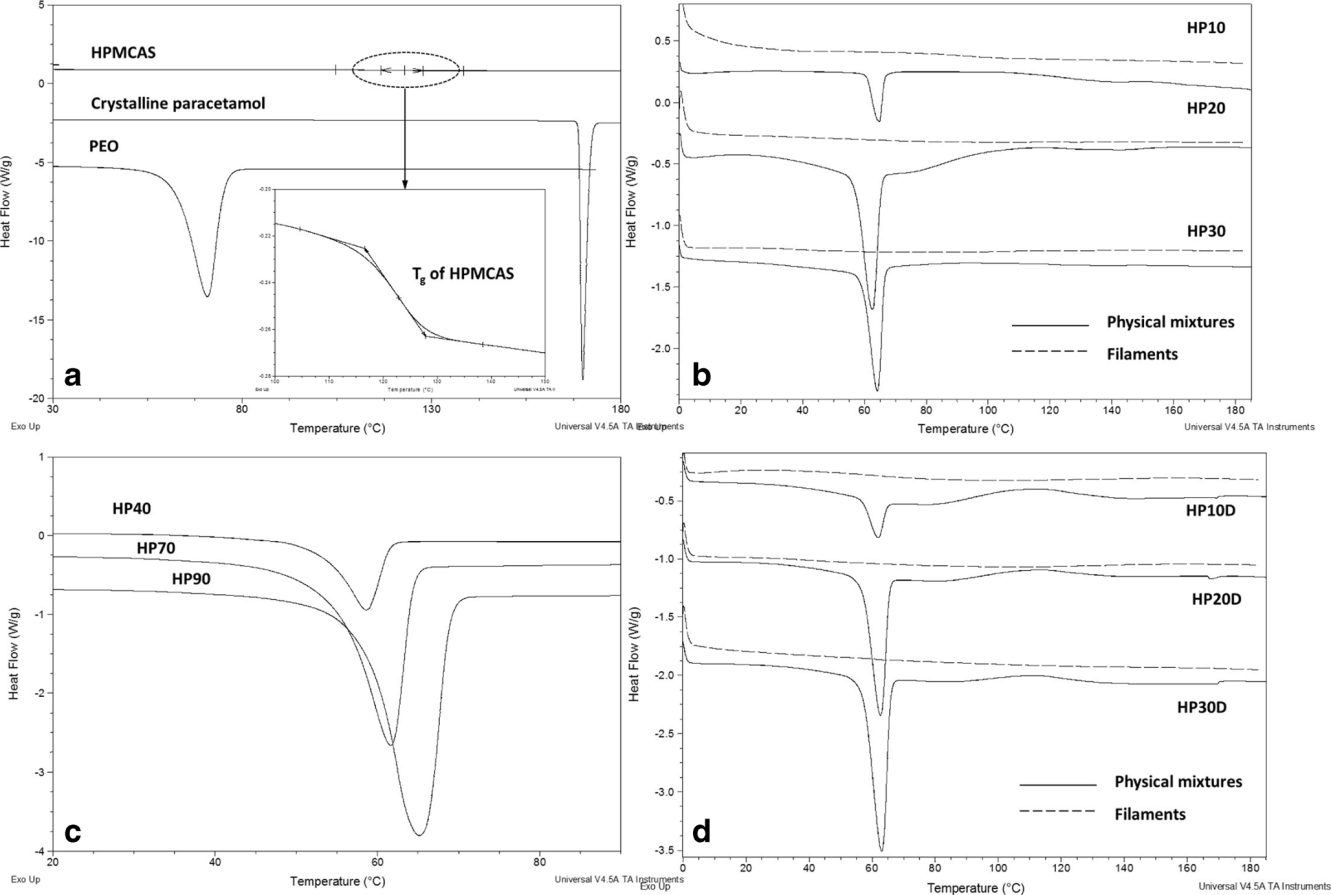
DSC thermograms of (**a**) raw materials of the HP filaments, (**b**) placebo HP filaments (dashed lines) and physical mixes (solid lines), (**c**) placebo fialments with high PEO loadings; and (**d**) drug loaded HP filaments (dashed lines) and physical mixes (solid lines).

There are 3 categories of pharmaceutical filaments produced and tested in this study; pure pharmaceutical polymers which are hot melt extrudable (HPMCAS, PEO, PVPVA 64, Soluplus, Eudragit EPO), polymer-plasticizer blends (HPMCAS-PEO, EUD, Soluplus-PEG, Soluplus-Tween), and drug loaded filaments (HPMCAS-PAC, HPMCAS-PEO-PAC). The rationale of including these different categories of the pharmaceutical filaments is to test a broad range of pharmaceutical materials to validate the screening method and develop understandings of the effects of additives and drugs on the feedability and printability of the pharmaceutical polymers. Figure 2 shows the DSC thermograms of the physical mixes and extruded filaments of HPMCAS based filaments. The DSC results of the rest of the filaments are available in the Supplementary Materials Fig. S1 and the T_g_ of the filaments that were detectable by DSC are summarized in Table II. Melting endotherms corresponding to the T_m_ of PEO at ~60°C and T_g_ of HPMCAS at ~120°C were seen in all physical mixes of the placebo and drug loaded HPMCAS-PEO blends. For drug loaded physical mixes, a small melting endotherm at ~169°C can be seen corresponding to the melting of PAC form I (monoclinic form) (14). For placebo HP filaments, the melting of PEO is absent in the low-loading formulations (HP10, HP20, and HP30), but is clearly seen in formulations with PEO content above 30% *w*/w (HP40, HP70 and HP90). This result indicates that with 10–30% PEO loading, HPMCAS mixed well with PEO after extrusion and the significant amount of HPMCAS was sufficient to prevent PEO from recrystallization. When increasing the PEO content to above 30%, clear phase separation of crystallised PEO and HPMCAS-PEO phase can be identified. Using the melting enthalpy values of the PEO melting in the HP filaments and the enthalpy value of the pure PEO (obtained from the DSC results of pure PEO), it is possible to estimate the degree of crystallinity of PEO in the filaments. HP90 and HP70 have 56.3 and 51.5% crystallinity, respectively, which is much higher than the 30.8% for HP40. This indicates that in high PEO content filaments (HP70 and HP90) the continuous phase is the semi-crystalline PEO. This contrasts with low PEO content filaments (HP10–30) that has the HPMCAS as the continuous phase. For HP40, as the contents of HPMCAS and PEO are close, it is reasonable to expect that there is no one polymer dominates as a continuous phase, which would contribute to the significant mechanical property difference observed later in the texture analysis tests. For drug loaded HP30D a small melting endotherm of PEO was detected indicating the semi-crystalline nature of PEO in this drug-loaded filament. The T_m_ of PAC was not seen in any of the drug-loaded filaments, suggesting no crystalline PAC in the filaments. The T_g_ values of the HP placebo and drug-loaded filaments could not be clearly identified.

**Table II:** Measured and Predicted T_g_’s of the Filaments

Formulation	T_g_ (°C)
HPMCAS	124
PEO	−60
PVP/VA 64	108
Soluplus®	70
Eudragit® EPO	45
HD	83
SP	42

*The T_g_ of the rest of the in-house filaments were not clearly identified using DSC

**Fig. 3:**
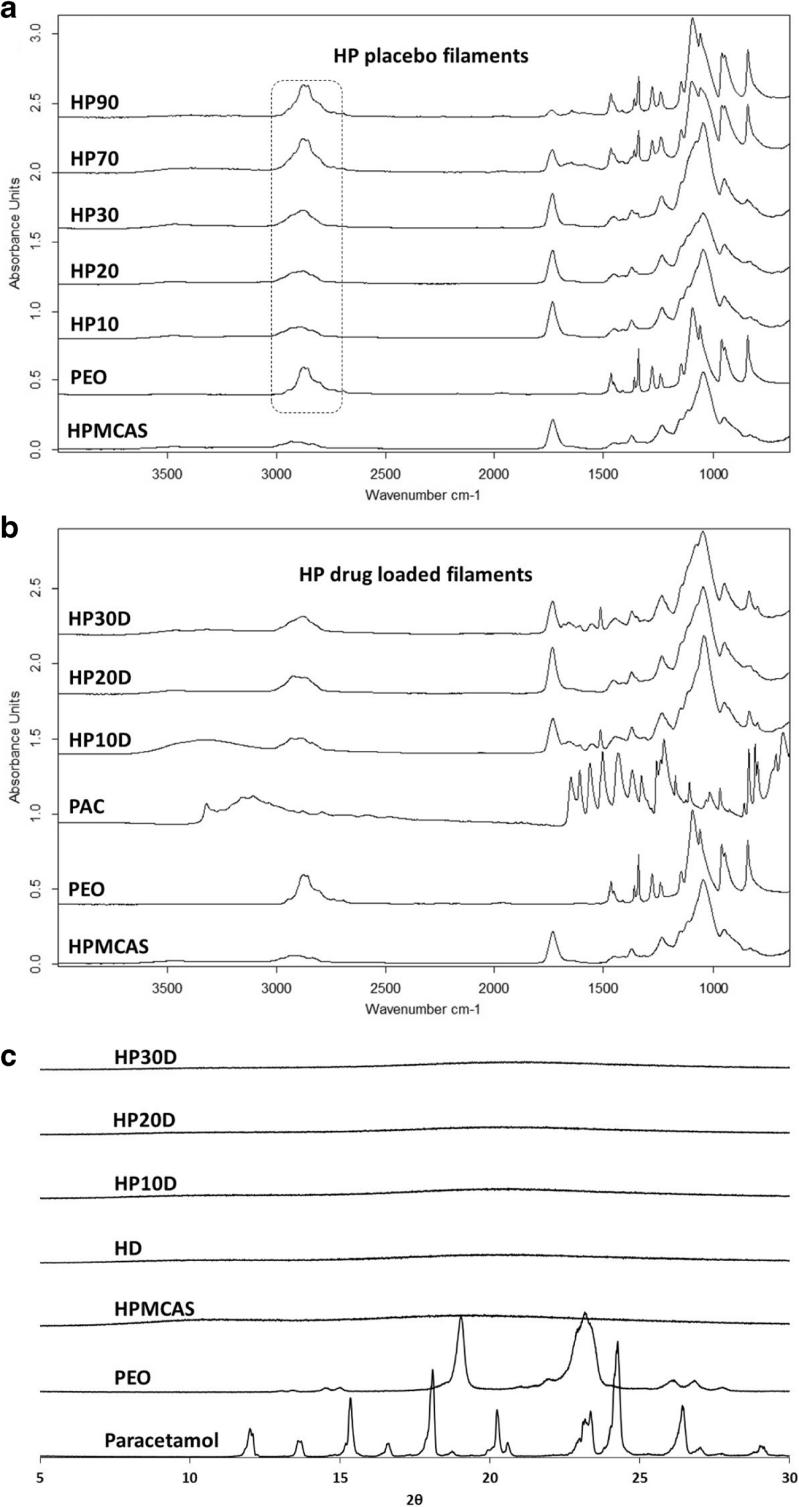
ATR-FTIR spectra for HP (**a**) placebo filaments, (**b**) drug loaded filaments, and (**c**) PXRD diffraction patterns of the drug loaded filaments.

ATR-FTIR and PXRD were carried out to further confirm the amorphous nature of the filaments and investigate any possible molecular interactions between the polymers and additives. Figure 3a shows the ATR-FTIR spectra of PEO and HPMCAS, as well as mixture filaments. The spectra of HP10 and HP20 closely resemble that of HPMCAS. The C-H stretching peaks of semi-crystalline PEO, occurring at 2876 cm^−1^ can be seen slightly more defined in HP30, At higher PEO concentrations the sharp PEO bands are dominating, nearly resembling the raw PEO material, and indicating significantly increased crystallinity of PEO in these filaments. In the spectra of HP70 and HP90 the PEO peaks at 1341 and 1077 cm^−1^, corresponding to C-H and O-H bending, are visibly unchanged in the placebo filaments indicating no specific interactions between HPMCAS and PEO. Figure 3b shows the ATR-FTIR spectra of PAC loaded filaments. Across the whole spectrum, the sharp bands of crystalline PAC are absent and a broad peak at 3321 cm^−1^, which corresponds to the N-H stretching of PAC in its amorphous state, can be seen in all drug-loaded filaments. In the drug containing samples, there was no observable change in the carbonyl peak of HPMCAS indicating the no significant hydrogen bonding interactions with the drug. The PXRD patterns of the filaments shown in Fig. 3c confirm the fully amorphous nature of all drug-loaded filaments, as neither PEO nor PAC signals are found in the diffraction patterns of HP10D, HP20D, and HP30D filaments.

### Filament Feedability Tests

Pure polymer filaments Eudragit® EPO, HPMCAS, PVP/VA 64, and Soluplus® were found to be too brittle and would fracture inside the printing head whenever feeding was attempted. With the addition of low levels (10% *w*/w) of drug (PAC) or plasticiser (PEG), HD and SP could still not be fed through the FDM printer. Increasing the degree of the plasticization (Tween 80 20% for ST and PEO 40% for HP40) in the filaments led to over-plasticization. The ST and HP40 filaments were found to be overly flexible and coiled up inside the feeding zone and would not thread through into the melting zone of the printing head. The rest of the pharmaceutical filaments were successfully fed through the FDM printer. The feedability test results are summarized in Table III.

**Table III:** Feedability and Correlation Coefficients of the Flexibility Profiles of In-House Pharmaceutical Filaments with the Flexibility Profiles of the Commercial Filaments

Filament	Feedability	ABS	Dissolvable filament	PLA	Average score	Rounded mean correlation score
HPMCAS	N	0.38	0.64	0.18	0.40	0
Mowiflex	Y	0.76	0.69	0.74	0.73	1
PEO	Y	0.92	0.71	0.82	0.82	1
PVPVA64	N	−0.08	0.22	−0.12	0.01	0
Soluplus	N	−0.56	0.40	−0.75	−0.30	0
Eudragit EPO	N	−0.70	0.30	−0.79	−0.40	0
EUD	Y	0.94	0.65	0.88	0.82	1
HD	N	0.32	0.60	0.18	0.37	0
HP10	Y	0.85	0.82	0.67	0.78	1
HP10D	Y	0.96	0.53	0.94	0.81	1
HP20	Y	0.94	0.70	0.87	0.84	1
HP20D	Y	0.90	0.48	0.95	0.78	1
HP30	Y	0.95	0.77	0.89	0.87	1
HP30D	Y	0.91	0.40	0.94	0.75	1
HP70	Y	0.70	0.73	0.51	0.65	1
HP90	Y	0.81	0.81	0.74	0.79	1
SP	N	0.49	0.63	0.28	0.47	0
Correlation matrix of the three commercial filaments						
	ABS		Dissolvable filament	PLA		
ABS	1		–	–		
Dissolvable Filament	0.62		1	–		
PLA	0.92		0.5	1		

### TA Screening Tests

**Fig. 4:**
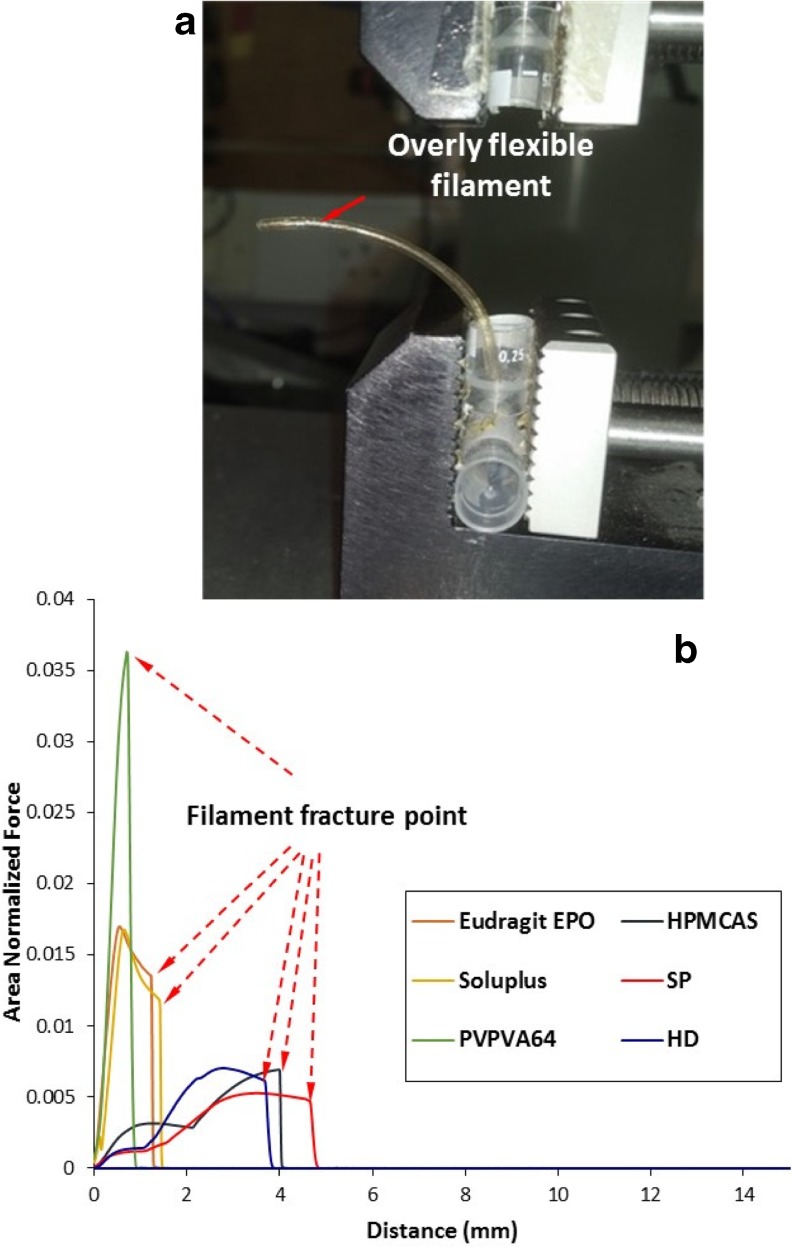
(**a**) Image of an example of a floppy filament (HP40) which was not able to be fed into the texture analysis rig for testing; (**b**) Texture analysis profile of non-feedable filaments.

**Fig. 5:**
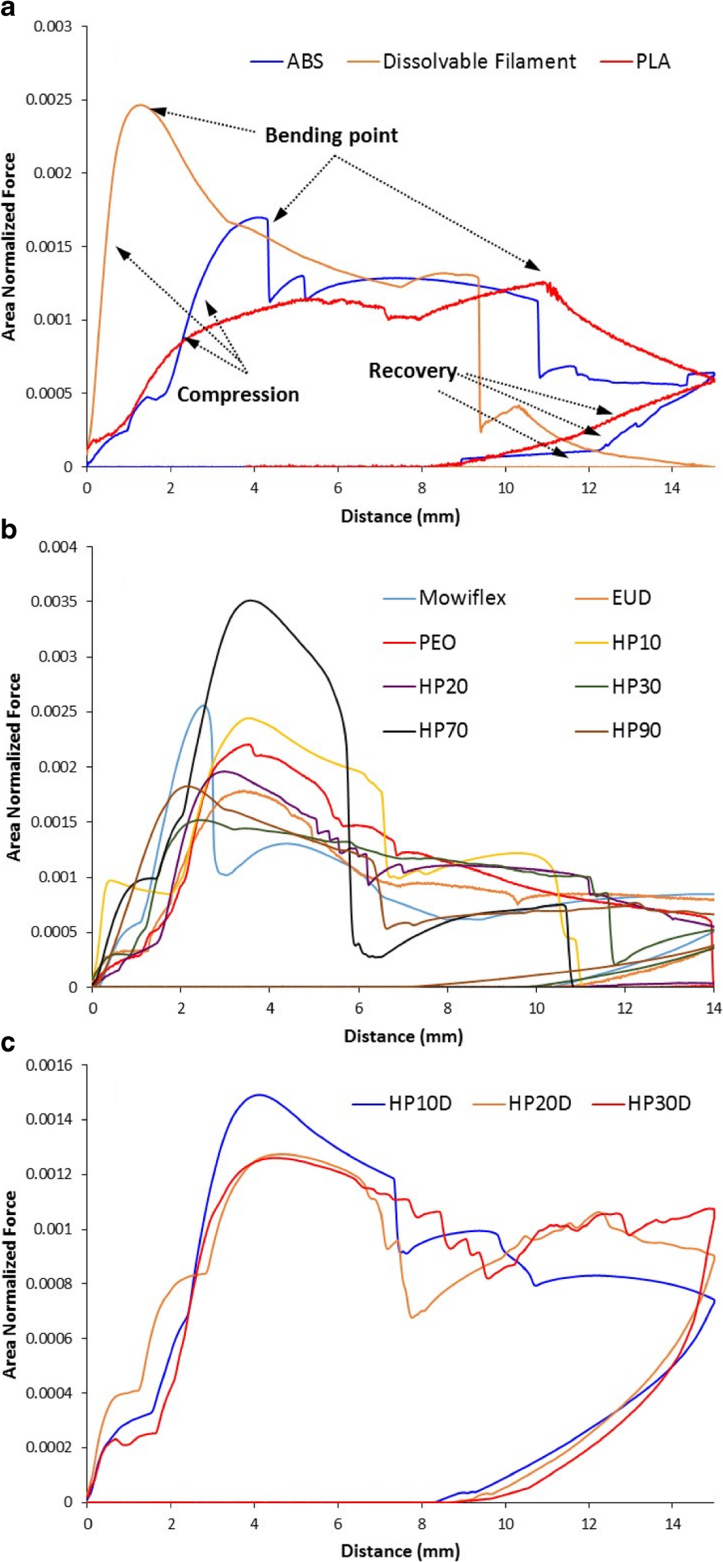
Flexibility profiles of feedable filaments. (**a**) the commercial filaments, (**b**) the placebo HP filaments, (**c**) PAC-loaded HP filaments.

TA tests were used to obtain the flexibility profiles of the filaments under axial compression. The areas under the curves were then normalized and plotted to give the comparisons of stress on the filament *vs*. distance travelled by the probe as seen in Figs. 4 and 5. Using the data, it is possible to group their behaviour to examine the correlation between the flexibility profile and the feedability of the filaments tested directly using the FDM printer. The ST and HP40 filaments were too flexible to be placed in the TA rig (as illustrated in Fig. 4a). The rest of the non-feedable filaments all shared a characteristic brittle fracture pattern, with sudden discontinuation of force on the filament after reaching a peak fracture force, as seen in Fig. 4b. These filaments fractured immediately at the maximum force, showing no bending, plastic, or elastic deformation to accommodate the increased strain. Within the non-feedable filaments, Eudragit EPO, PVPVA64 and Soluplus exhibited much sharper fracture than HPMCAS, HD and SP. This is evident by the longer travel distance of the probe before the fracture of the HPMCAS, HD and SP filament, and by the existence of some minor resistance to deformation by the filaments after reaching the yielding force.

**Fig. 6:**
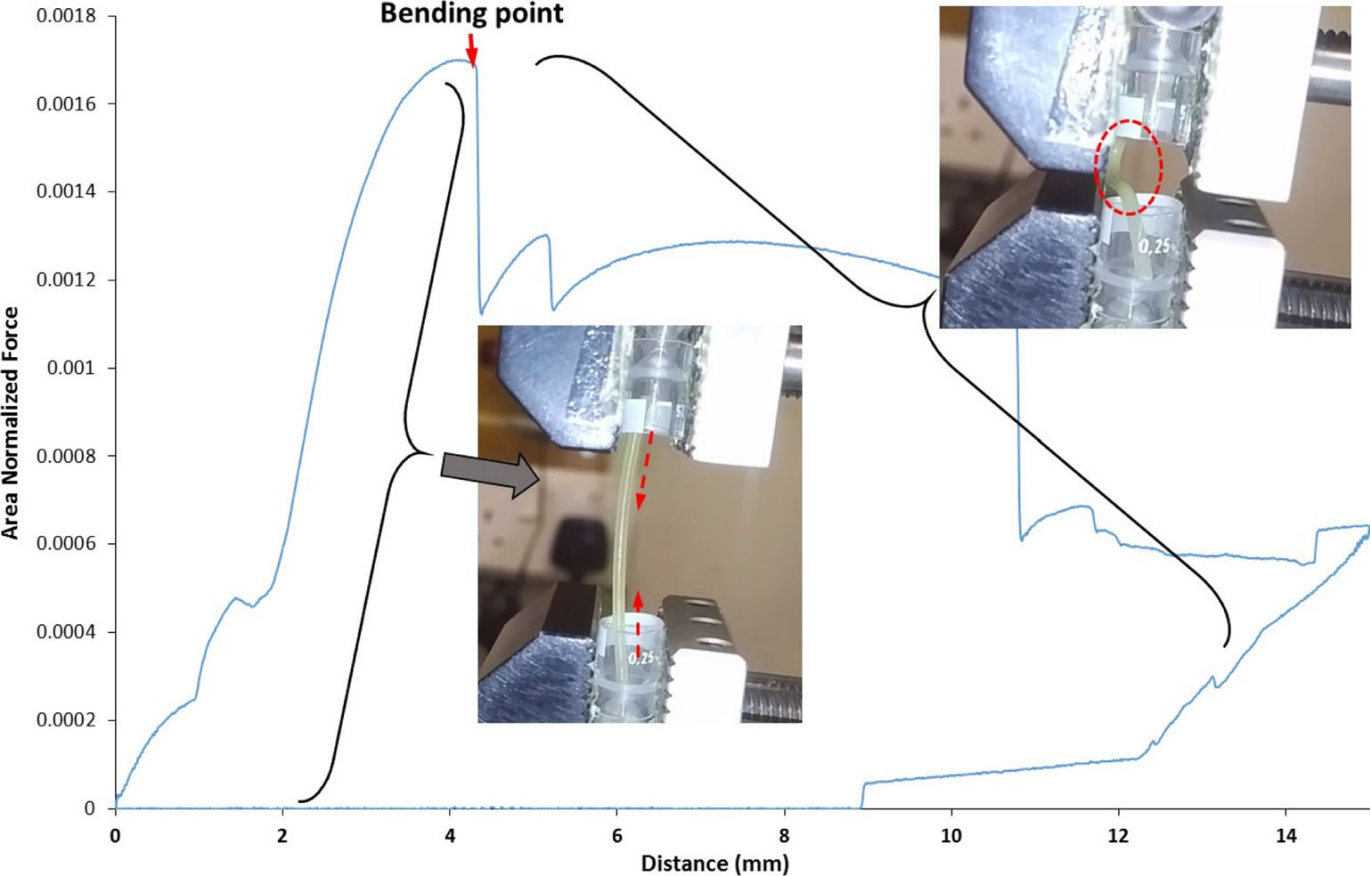
Illustration of the interpreation of the force-distace of a feedable filament during TA.

Despite seeming random at first glance, the flexibility profiles of the feedable filaments, as seen in Fig. 5, all share a characteristic bending deformation after the maximum strain bearing point is reached. When the TA probe was returning to the start position, the filaments could partially recover and straighten within the rig even though they had lost some of their stiffness. Of the feedable filaments, filament HP10 was notable the only filament to fracture during TA. However, filament HP10 did exhibit substantial bending after reaching peak tension force and only fractured after being bent considerably by the texture analyzer probe. Therefore, its recorded fracture pattern was found to considerably differ from the sharp brittle fracture patterns exhibited by non-feedable filaments, mainly HD and SP. Figure 6 shows an example of a flexibility profile combined with photographs of the filament at critical points in the profile. During TA testing, the filament is subjected to axial compression forces, as the TA probes continue moving towards each other, the forces born by the filament continuously increase. At the first critical point, the filament reaches the Euler point (yield point), above which even the application of an infinitesimal lateral force will cause bending. This is the bending point highlighted in Fig. 6. After this, the applied force acts to both compress the filament and to further bend the filament at the weakened bend point which leads to the complex TA profile pattern of this stage of test as seen in Figs. 5 and 6. Notably, this characteristic feature of bending and maintaining structural integrity above the Euler point was seen in all feedable filaments as shown in Fig. 5.

### Correlation Analysis

The correlations between the flexibility profiles (the normalized area under the flexibility profile) of each in-house filaments and each of the three commercial FDM printable filaments, ABS, dissolvable filament and PLA, were generated and listed in Table III. This correlation can be treated as the quantification of the level of similarity between the flexible profile of the in-house pharmaceutical filaments and the commercial printable filaments. The higher the correlation score, the higher the mechanical similarity of the tested filament to the commercial filaments. As seen in Table III, for most of the filaments, the correlation scores to the 3 commercial filaments vary. This is not surprising as the correlations scores of the flexible profiles of the 3 commercial filaments also varies indicating there are some differences in their flexible profiles. The correlation scores were further analysed by taking the mean of the three correlation scores per filament using the equation

2 $$ \frac{C_{ABS}+{C}_{Dissolvable\ Filament}+{C}_{PLA}}{3} $$

where C_x_ is the correlation score with commercial filament x. Overall, all the in-house filaments that passed the feedability test had an average correlation score above 0.5; whereas the filaments that failed the feedability test all had a correlation score below 0.5. Furthermore, the correlation scores of plasticized filaments were higher than those of non-plasticized filaments indicating that plasticization improves the flexibility of the filaments.

### Principal Component Analysis (PCA)

PCA is a multivariate statistical technique that, from a data table containing observations describing a multitude of inter-related variables, can extract key information which is represented as functions of “Principal Components”. Similarities between the observations can be represented by plotting the variables on a map referred to as a *space plot* (15). PCA was performed to further explore the relationship between flexibility profile of the filaments and their feedability. As the correlation scores of the Dissolvable filament with the other commercial filaments are low, the dissolvable filament was treated as an outlier and was not included in the PCA. For the PCA, the normalized full force-distance curves were used the analysis. Principal Component 1 shows an eigenvalue of 10.13, Principal Component 2 shows an eigenvalue of 3.57, Principal Component 3 shows an eigenvalue of 1.51, while all other principal components show an eigenvalue <1. By applying the Kaiser Rule, the first three principal components were extracted and the component loadings are shown in Supplementary Materials Table S1.

**Fig. 7:**
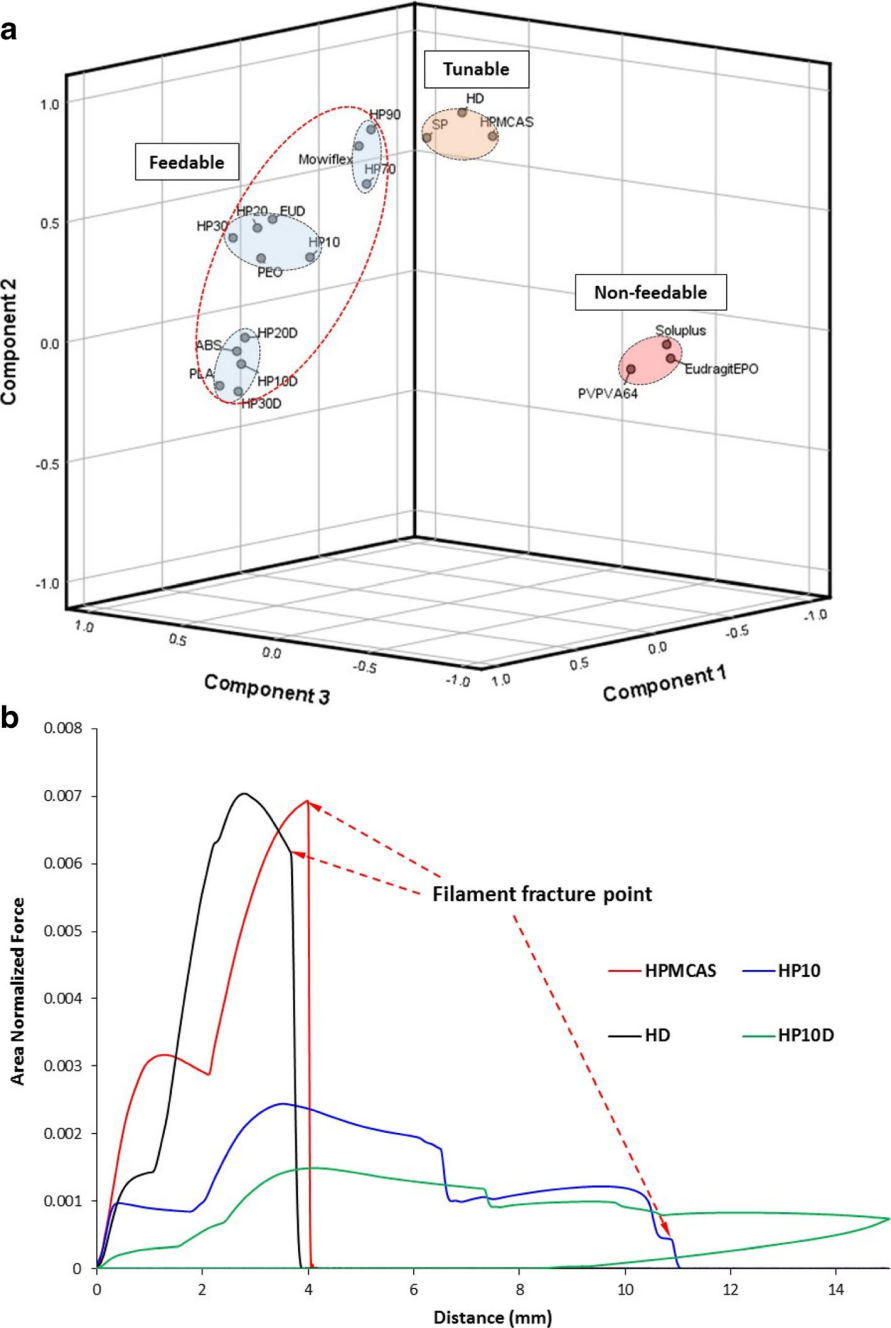
(**a**) 3-dimensional rotated space plot of principal components 1, 2 and 3; (**b**) comparison of the flexible profiles of non-feedable, tunable and feedable HPMCAS based filaments.

Figure 7a shows the rotated space plot of Principal Components 1, 2, and 3. The filaments aggregated on the plot into five clusters. The feedable filaments aggregated into three clusters, the first containing ABS, PLA, HP10D, HP20D, and HP30D. The second contains filaments HP10, HP20, HP30, EUD, and PEO. The third cluster contains filaments Mowiflex, HP70, and HP90. The three clusters are closely aggregated together and can be looked at as a single macro-cluster.

The fourth cluster contained filaments SP, HD, and HPMCAS, which are the filaments that showed some strain-bearing ability in the TA tests but still fractured as the result of compression (Fig. 4b). This cluster of slightly deformable brittle filaments is closely positioned to the macro-aggregated cluster of feedable filaments. Although the filaments in this cluster are not feedable, they exhibited some potential, such that, with formulations modification such as adding plastisisers, they can be possibly be tuned to become feedable. The transformation of the mechanical properties of HPMCAS upon the addition of a drug (PAC) and a second polymer (PEO) is an example of such tuning (Fig. 7b). The fifth cluster contains the highly brittle and non-feedable filaments Eudragit® EPO, PVPVA 64, and Soluplus^®^.

## Discussion

### Correlation between Mechanical Properties and Feedability

This study is aimed to develop a screening method to speed up the formulation development of FDM printable solid formulations. To achieve good printability, the FDM filaments first need to exhibit good feedability to allow the smooth and continuous delivery of the filaments to the melting zone of the printing head. As previously discussed, the feeding mechanism employed by FDM printers involves mechanical pushing of a filament held between two counter-rotating gears. In situations where the filament being fed is too brittle to bear the mechanical strain generated from the compression and pushing, this is likely to cause the filament to fracture, discontinuing the force that is propelling the filament forward, causing a block in the printing head. If it is too ductile it will deform when begin passed forward and again block the head.

For developing pharmaceutical FDM filaments, the polymers firstly must be hot melt extrudable to form the filaments. When HME is used in the fabrication of traditional solid dosage forms (i.e. tablets), the extrudates produced often undergo particle size reduction, to produce powders or granules with a suitable particle size for pharmaceutical processing (i.e. compression) (16). Unsurprisingly, brittle extrudates are more suitable in that regard, as brittle materials require less time and energy to be milled or granulated as opposed to ductile/flexible materials (17). Therefore, most pharmaceutically relevant polymers that are suitable for HME often yield brittle extrudates that readily fracture, and while this makes such polymers suitable for traditional pharmaceutical applications of HME, it renders those polymers unsuitable for FDM implementation.

TA studies clearly show that there exists two types of non-feedable filaments, both highly friable, either with or without any strain bearing ability. The additions of plasticizers caused an increase in the strain bearing ability of the filaments. As an example, pure Soluplus filament exhibited no strain bearing capacity in the TA test, whereas the addition of 10% PEG (SP) shifted the flexibility profile to the group exhibiting some strain bearing capacity, owing to the plastisisation effect of the PEG. The incorporation of plasticizers also decreases the glass transition, and improves melt flow properties of thermoplastic polymers, which are important factors that influence FDM. Among the plasticizers used in the formulation of pharmaceutical blends for FDM printing are triethyl citrate, triacetin, various grades of polyethylene oxides (PEG and PEO), Tween® 80, and glycerol (6,8–11). However when comparing the T_g_ data in Table II and the feedability data in Table III, it is clear that the absolute T_g_ values of the filament do not directly correlate to their feedability.

Over-plasticization of filaments was observed to also cause a feeding defect; filaments HP40 and ST were found to coil inside the printing head when feeding was attempted. Those filaments possess little-to-no rigidity and would readily deform when any force is applied, with their texture being more like that of fabrics than thermoplastic polymers. This indicates that the appropriate level of plasticization is vitally important. The non-feedability of the overplasticized filaments is because they readily deform inside the printing head making them unable to thread through the melting zone for deposition. This lack of rigidity sits in stark contrast to feedable filaments which are pliable enough to bend and deform on handling, but retain their original shape when force is released.

HPMCAS was selected as the platform polymer for drug loading and plasticization screening because, although the filament itself was not feedable, its flexibility profile clearly displayed some strain-bearing properties comparable to those of plasticized Soluplus (SP). The addition of 10% PEO was found to readily transform the filament into a feedable one. Furthermore, the addition of 10% PAC to the filament significantly changed its fracture pattern from a brittle fracture to a slightly more ductile fracture (Fig. 7b). This can be attributed to the plasticization effect of the drug on the polymer. This is also supported by the fact that although HP10 did fracture (but was still feedable), filament HP10D did not, suggesting that the addition of 10% PAC further increased the strain-bearing ability of the filament HP10.

Filaments HP10, HP20, HP30, HP20D, and HP30D were all found to be feedable, suggesting that, at least for HPMCAS, there is a wide margin available for plasticizer loading without unduly influencing the mechanical properties. Increasing the PEO loading to 40% rendered the filament unfeedable (HP40), which most likely is due to the significant phase separation of HPMCAS and PEO as indicated by the DSC in Fig. 2. Conversely, filaments HP70 and HP90 were found to be feedable. However, the high PEO loading in comparison to HP10, HP20, and HP30 means that the filaments are most likely PEO-based with the HPMCAS being the second material in the matrix. This is further supported by the color difference; HP10, HP20, and HP30 all had the characteristic pale yellow color of hot-melt extruded HPMCAS (18), while filaments HP70 and HP90 were colored identically to the PEO filament. Based on these results, it is reasonable to hypothesize that, in the case of HPMCAS-PEO blends, the existence of a continuous phase (either HPMCAS or PEO) as the primary matrix former is important to maintain the mechanical strength of the filaments.

### Using Flexibility Profile towards Screening

In terms of screening, the TA test was designed to simulate the conditions inside the printing head as closely as possible, the speed of compression was set to 3.15 mm/s, which matches the speed of feeding inside the printer. Tested filaments were found to exist in either one of three categories; brittle filaments, string-like filaments, and pliable filaments. Brittle filaments are filaments that fracture during the analysis. String-like filaments are filaments could not be tested due to them being too flexible to maintain a vertically suspended straight beam shape and would collapse under their own weight. Pliable filaments are filaments that would deform due to compression by the texture analyzer, but recover when the force is removed.

It should be noted that the aforementioned categories do not have clearly defined boundaries, but are rather like a spectrum. Filaments SP, HD, and HPMCAS, despite being brittle filaments, did exhibit some pliability before fracturing. Inversely, filament HP10 did fracture during TA, but the predominating mechanical property it exhibited was pliability.

High correlation scores between the feedable in-house filaments and the commercial filaments were observed. All feedable filaments displayed correlation scores >0.50 with the commercial filaments, indicating the significance of the relationship between the flexibility profiles of the filaments and their feedability. However, no observations were made that indicate feedability existing as a spectrum property of the filaments (i.e. no filaments were found to be “more feedable than others”). Feedability of the filaments is a Boolean value, being either true of false. The TA data is a curve which is then normalized and sorted into categories by the statistical analysis. The normalisation procedure used in this removes differences in the absolute values of force applied. Mowiflex is feedable but notably much stiffer than all other tested materials, with the minimum required force for deforming Mowiflex being 120N. The higher stiffness of the filament does not affect its feedability and indicates that a range of absolute mechanical properties might lead to feedable materials provided the shape of the overall flexibility profile falls within the acceptable range. To further simplify the analysis, rounding the mean correlation scores of each filament to the nearest integer (values <0.5 are rounded down to 0, while values >0.5 are rounded to 1) produces a method (Table III) that simply sorts the filaments into feedable with a score of 1 (True) and non-feedable filaments with a score of 0 (False).

PCA was used as a qualitative statistical method to sort the different filaments using their flexibility profiles into feedable and non-feedable filaments. As seen in Fig. 7a, three clusters were observed in the rotated space plot of the filament flexibility profiles. The feedable and non-feedable filaments are well separated. Interestingly a cluster containing filaments that can be easily tuned to become feedable (referred in the Fig. 7a as ‘tunable’ filament) is also isolated. Using HPMCAS as an example (Fig. 7b), by adding different types and amounts of plasticizers, the non-feedable polymer and polymer blend (HPMCAS and HD) in this cluster can be transferred into feedable filament (HP10 and HP10D). This data demonstrates that the flexible profile obtained from TA test can be correlated to the feedability and used to predict the potential of the FDM printability of the targeted materials.

## Conclusion

Mechanical properties of the HME filaments is an important property determining the processibility for FDM 3DP. By measuring the flexibility, one of the most directly relevant mechanical properties of the HME filaments, this study described the development of a simple method for screening the feedability and subsequent printability of HME filament for FDM printing. A wide range of filaments prepared using pharmaceutical polymers and excipients were tested to validate the method. The method described could accurately and reproducibly separate feedable and non-feedable filaments. Furthermore, coupled with PCA, more insights were gained in the aspects of how plasticisation and phase separation could influence the feedability of the pharmaceutical filaments.

## Electronic supplementary material

ESM 1 (DOCX 578 kb)

## Abbreviations

3DP: 3D printing
ABS: Acrylonitrile butadiene styrene
ATR-FTIR: Attenuated Total Reflectance Fourier Transform Infrared
CPP: Critical processing parameters
DSC: Differential Scanning Calorimetry
FDA: Food and Drug Administration
FDM: Fused Deposition Modeling
HME: Hot-Melt Extrusion
HPC: hydroxypropyl cellulose
HPMCAS: Hypromellose acetate succinate
PAC: Paracetamol
PCA: Principal Component Analysis
PEG: Polyethylene glycol
PEO: Polyethylene glycol
PLA: Polylactic acid
PVA: Polyvinyl alcohol
PXRD: Powder X-Ray Diffraction
TA: Texture Analysis

## References

[1] Norman J, Madurawe RD, Moore CMV, Khan MA, Khairuzzaman A (2017). A new chapter in pharmaceutical manufacturing: 3D-printed drug products. Adv Drug Deliv Rev.

[2] Alhnan MA, Okwuosa TC, Sadia M, Wan KW, Ahmed W, Arafat B (2016). Emergence of 3D printed dosage forms: opportunities and challenges. Pharm Res.

[3] Bakar NSA, Alkahari MR, Boejang H (2010). Analysis on fused deposition modelling performance. J Zhejiang Univ Sci A.

[4] Mohamed OA, Masood SH, Bhowmik JL (2015). Optimization of fused deposition modeling process parameters: a review of current research and future prospects. Adv Manuf.

[5] Jin YA, Li H, He Y, Fu JZ (2015). Quantitative analysis of surface profile in fused deposition modelling. Addit Manuf.

[6] Goyanes A, Buanz ABM, Basit AW, Gaisford S (2014). Fused-filament 3D printing (3DP) for fabrication of tablets. Int J Pharm.

[7] Goyanes A, Buanz ABM, Hatton GB, Gaisford S, Basit AW (2015). 3D printing of modified-release aminosalicylate (4-ASA and 5-ASA) tablets. Eur J Pharm Biopharm.

[8] Skowyra J, Pietrzak K, Alhnan MA (2015). Fabrication of extended-release patient-tailored prednisolone tablets via fused deposition modelling (FDM) 3D printing. Eur J Pharm Sci.

[9] Pietrzak K, Isreb A, Alhnan MA (2015). A flexible-dose dispenser for immediate and extended release 3D printed tablets. Eur J Pharm Biopharm.

[10] Alhijjaj M, Belton P, Qi S (2016). An investigation into the use of polymer blends to improve the printability of and regulate drug release from pharmaceutical solid dispersions prepared via fused deposition modeling (FDM) 3D printing. Eur J Pharm Biopharm.

[11] Melocchi A, Parietti F, Maroni A, Foppoli A, Gazzaniga A, Zema L (2016). Hot-melt extruded filaments based on pharmaceutical grade polymers for 3D printing by fused deposition modeling. Int J Pharm.

[12] Huang T, Wang S, He K (2015). Quality control for fused deposition modeling based additive manufacturing: current research and future trends. 2015 First Int Conf Reliab Syst Eng.

[13] Stokes M. 3D printing for architects with MakerBot : build state-of-the-art architecture design projects with MakerBot replicator 1, 2, or 2X. Packt Pub; 2013.

[14] Boldyreva EV, Drebushchak VA, Paukov IE, Kovalevskaya YA, Drebushchak TN (2004). DSC and adiabatic calorimetry study of the polymorphs of paracetamol: An old problem revisited. J Therm Anal Calorim.

[15] Williams LJ (2010). Principal component analysis. Wiley Interdiscip Rev Comput Stat 2.

[16] Maniruzzaman M, Boateng JS, Snowden MJ, Douroumis D (2012). A review of hot-melt extrusion: process technology to pharmaceutical products. ISRN Pharm.

[17] Iyer RM, Hegde S, DiNunzio J, Singhal D, Malick W (2014). The impact of roller compaction and tablet compression on physicomechanical properties of pharmaceutical excipients. Pharm Dev Technol.

[18] Lu J, Obara S, Ioannidis N, Suwardie J, Gogos C, Technical C (2016). Understanding the processing window of hypromellose acetate succinate for hot-melt extrusion, part I: polymer characterization and hot-melt extrusion. Adv Polym Technol.

