# Supplementary material for: Development of a Simple Mechanical Screening Method for Predicting the Feedability of a Pharmaceutical FDM 3D Printing Filament
Source: Pharm Res. 2018 May 31;35(8):151. doi: 10.1007/s11095-018-2432-3 (PMC5982458; doi:10.1007/s11095-018-2432-3)
Supplement: Supplementary file 1 — (DOCX 578 kb) [file 11095_2018_2432_MOESM1_ESM.docx]

**Supplementary Material**

Development of a simple mechanical screening method for predicting the feedability of a pharmaceutical FDM 3D printing filament

Jehad M. Nasereddin^1^, Nikolaus Wellner^2^, Muqdad Alhijjaj^1,3^, Peter Belton^4^, Sheng Qi^1^*

^1^School of Pharmacy, University of East Anglia, Norwich, Norfolk NR4 7TJ, United Kingdom;

^2^Quadram Institute Bioscience, Norwich Research Park, Colney, Norwich, Norfolk, NR4 7UA, United Kingdom

^3^Department of Pharmaceutics, College of Pharmacy, University of Basrah, Basrah, Iraq

^4^School of Chemistry, University of East Anglia, Norwich, Norfolk NR4 7TJ, United Kingdom

* Corresponding Author. Email address: [sheng.qi@uea.ac.uk](mailto:sheng.qi@uea.ac.uk) (S. Qi)

Table S1: Loadings of the first three principal components of the PCA analysis

|  | Component 1 | Component 2 | Component 3 |
| --- | --- | --- | --- |
| ABS | .848 | .412 | -.014 |
| Eudragit EPO | -.384 | -.877 | -.020 |
| HP20 | .749 | .415 | .474 |
| HPMCAS | .017 | -.209 | .885 |
| Mowiflex | .112 | .423 | .711 |
| EUD | .788 | .303 | .520 |
| PEO | .873 | .282 | .369 |
| Soluplus | -.411 | -.831 | .016 |
| PLA | .706 | .615 | -.221 |
| SP | .295 | -.088 | .875 |
| PVPVA64 | -.200 | -.823 | -.037 |
| HP10 | .875 | .017 | .386 |
| HP30 | .620 | .649 | .371 |
| HD | -.128 | .082 | .931 |
| HP10D | .943 | .304 | -.074 |
| HP20D | .872 | .348 | .000 |
| HP30D | .905 | .346 | -.187 |

**Figure S1.** DSC thermograms of the non-feedable filament
